# TOX expression in cutaneous B-cell lymphomas

**DOI:** 10.1007/s00403-016-1654-7

**Published:** 2016-05-14

**Authors:** Anne M. R. Schrader, Patty M. Jansen, Rein Willemze

**Affiliations:** Department of Pathology, Leiden University Medical Center, P.O. box 9600, 2300 RC Leiden, The Netherlands; Department of Dermatology, Leiden University Medical Center, Leiden, The Netherlands

**Keywords:** TOX, Cutaneous B-cell lymphoma, Primary cutaneous follicle center lymphoma, Primary cutaneous marginal zone lymphoma, Primary cutaneous diffuse large B-cell lymphoma, leg type, Immunohistochemistry

## Abstract

Thymocyte selection-associated high-mobility group box (TOX) is aberrantly expressed in cutaneous T-cell lymphomas. In a recent study, TOX expression was noted unexpectedly in the follicle center (germinal center) B-cells of reactive lymph nodes and tonsils, used as external controls. To evaluate whether TOX is also expressed by cutaneous B-cell lymphomas, TOX immunohistochemistry was performed on skin biopsies of 44 patients with primary and secondary cutaneous B-cell proliferations. TOX was expressed not only in the reactive follicle center cells of lymph nodes, tonsils, cutaneous lymphoid hyperplasia, and primary cutaneous marginal zone lymphomas, but also by the neoplastic follicle center cells of 16/17 patients with primary cutaneous follicle center lymphoma (PCFCL) and 7/7 patients with cutaneous manifestations of systemic follicular lymphoma (FL). Notably, TOX showed a very similar expression pattern as BCL6, a marker of germinal center B-cells. In 4/10 patients with a BCL6^+^ primary cutaneous diffuse large B-cell lymphoma, leg type (PCDLBCL,LT) and in 2/2 patients with a secondary cutaneous BCL6^+^ diffuse large B-cell lymphoma (DLBCL), TOX was expressed by more than 50 % of the neoplastic B-cells. In contrast, in 3/3 BCL6^−^ PCDLBCL,LT, TOX was completely negative or weakly expressed by a minor proportion of the neoplastic B-cells. In conclusion, TOX is expressed not only by neoplastic T-cells, but also by both reactive and neoplastic follicle center (germinal center) B-cells and a proportion of BCL6^+^ PCDLBCL,LT and secondary cutaneous BCL6^+^ DLBCL. The functional significance of TOX expression in reactive and neoplastic B-cells remains to be elucidated.

## Introduction

In the past few years, TOX [thymocyte selection-associated high-mobility group (HMG) box] has been described as a potential new diagnostic marker for cutaneous T-cell lymphoma (CTCL) [16]. TOX is a family member of HMG box proteins and is involved in the regulation of gene expression, as by modifying the density of the chromatine structure [13]. In general, TOX is associated with the development of CD4^+^ T-cells in the thymus and is subsequently downregulated in mature CD4^+^ T-cells [1]. TOX was also shown to be expressed in the development of other lymphoid cells, such as lymphoid tissue inducer (LTi) cells that normally act as key regulators of lymph node organogenesis [2].

Recent studies showed aberrant expression of TOX in different types of CTCL, mainly Sézary syndrome (SS) and mycosis fungoides (MF), but also in primary cutaneous CD30^+^ lymphoproliferative disorders, including primary cutaneous anaplastic large cell lymphoma and lymphomatoid papulosis (LyP), though with lower intensity, and cases of adult T-cell leukemia/lymphoma and peripheral T-cell lymphoma, not otherwise specified [4, 7, 8, 15]. Morimura et al. suggested that TOX expression is limited to CD4^+^ T-cells in MF and SS, and CD30^+^ T-cells in LyP [8]. However, in a recent study of our own group, TOX was also expressed by the neoplastic T-cells in the cases of MF and other CTCL with CD4^−^CD8^+^ or CD4^−^CD8^−^ phenotypes and by (intra-epidermal) CD8^+^ T-cells in the cases of atopic dermatitis [11]. Unexpectedly, expression of TOX was also noted in follicular areas of reactive lymph nodes and tonsils that were used as external controls. This prompted us to further study TOX expression in various types of cutaneous B-cell lymphoma (CBCL).

## Materials and methods

Formalin-fixed and paraffin-embedded (FFPE) skin biopsies from 44 patients with primary or secondary cutaneous B-cell proliferations were included: 17 patients with primary cutaneous follicle center lymphoma (PCFCL), 5 patients with primary cutaneous marginal zone lymphoma (PCMZL), 13 patients with primary cutaneous diffuse large B-cell lymphoma, leg type (PCDLBCL,LT), 7 patients with skin manifestation of systemic follicular lymphoma (FL), and 2 patients with skin manifestation of systemic diffuse large B-cell lymphoma (DLBCL). FFPE tissue samples of reactive lymph nodes and tonsils were included as benign external controls. In addition, two cases of cutaneous lymphoid hyperplasia (CLH; cutaneous pseudo B-cell lymphoma) were evaluated. All diagnoses were made by an expert panel of dermatologists and pathologists at one of the regular meetings of the Dutch Cutaneous Lymphoma Group, according to the classification system of the World Health Organization (WHO) and the European Organization for Research and Treatment of Cancer (EORTC) [14]. Therefore, sections from all biopsies were routinely hematoxylin and eosin (H&E) stained with additionally, depending on the differential diagnosis, a selection of the following immunostainings: CD3, CD4, CD5, CD8, CD10, CD20, CD21, CD23, CD30, CD35, CD68, CD79a, CD138, PAX5, BCL2, BCL6, MUM1, kappa, lambda, IgG, IgM, IgD, IgA, and/or Ki-67. In all patients with diagnosis of a primary CBCL, staging procedures excluded extracutaneous disease. For the purpose of this study, all FFPE skin biopsies were collected from the archives of the Department of Pathology, Leiden University Medical Center (LUMC), Leiden, The Netherlands, and immunohistochemistry for TOX was performed using Dako Autostainer Link 48 with rabbit anti-human TOX antibodies of Sigma-Aldrich (HPA018322) in a dilution of 1:200. The sections were evaluated by all authors, and the percentage of TOX^+^ cells was estimated until consensus was reached. The intensity of TOX expression was scored as dim or strong. The study was performed in accordance with the Declaration of Helsinki and the Dutch Code for Proper Secondary Use of Human Tissue, approved by the medical ethics committee of the LUMC.

## Results

### TOX expression in reactive lymph nodes, tonsils and cutaneous lymphoid hyperplasia

**Fig. 1:**
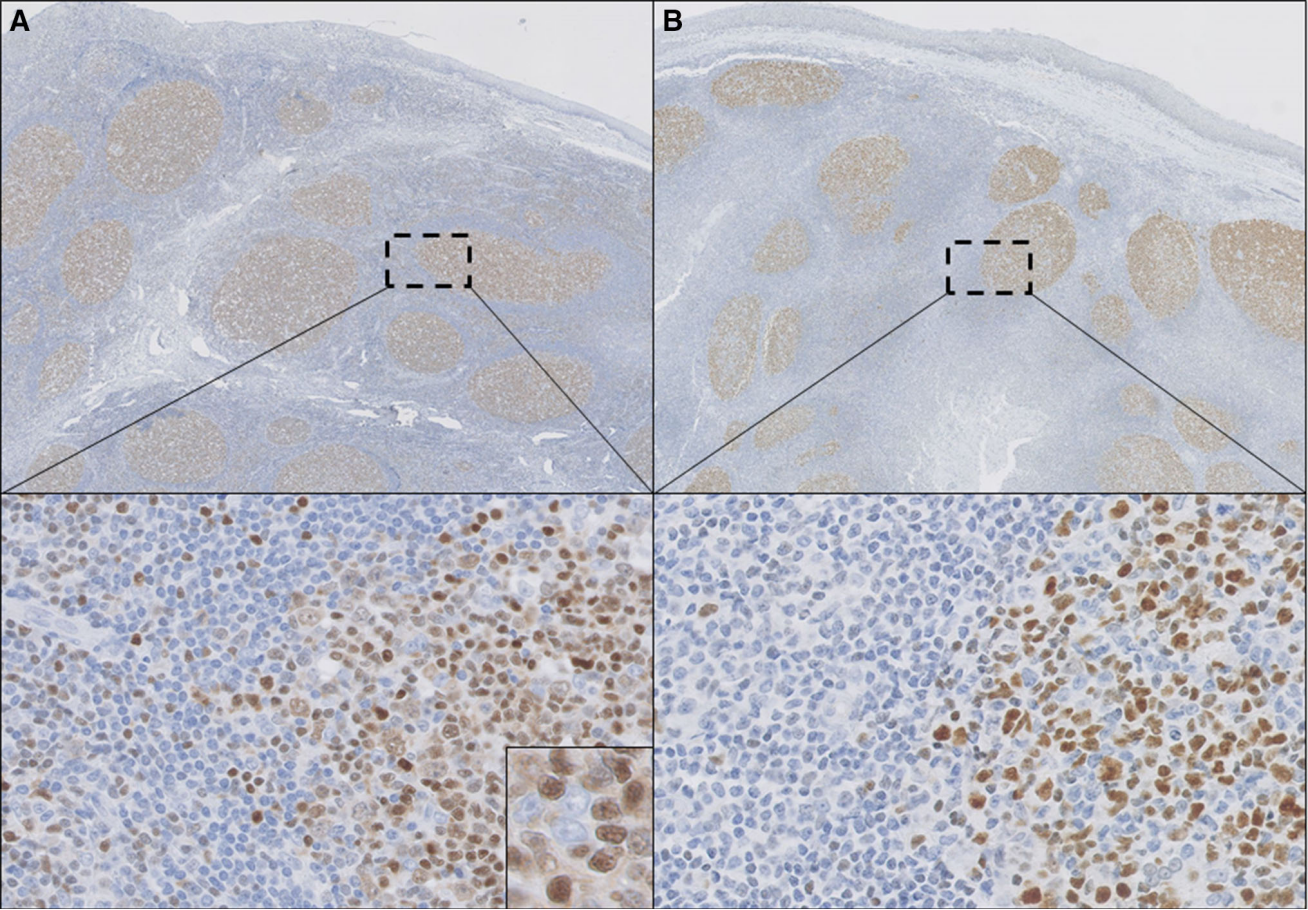
Histopathology of a tonsil with reactive follicles. TOX (**a**) is expressed in reactive follicles, predominantly in centrocytes (*inset*) in a pattern similar to BCL6 (**b**), but also in some scattered lymphocytes in the interfollicular areas (selected regions). (**a**, **b** ×100, selected regions in **a** and **b** ×200 and *inset* in **a** ×400)

In the reactive lymph nodes and tonsils that were used as benign external controls, TOX was expressed in the follicular areas and by scattered small lymphocytes, probably T-cells, in the interfollicular areas (Fig. 1). In the follicular areas, the centrocytes showed moderate to strong nuclear TOX expression, while staining of the centroblasts was dim or absent. Serial sections showed that the distribution of TOX^+^ cells was very similar to that observed for BCL6, a marker for germinal center B-cells. The same expression pattern was observed in both cases of CLH.

### TOX expression in primary cutaneous follicle center lymphoma and secondary cutaneous involvement of systemic follicular lymphoma

**Fig. 2:**
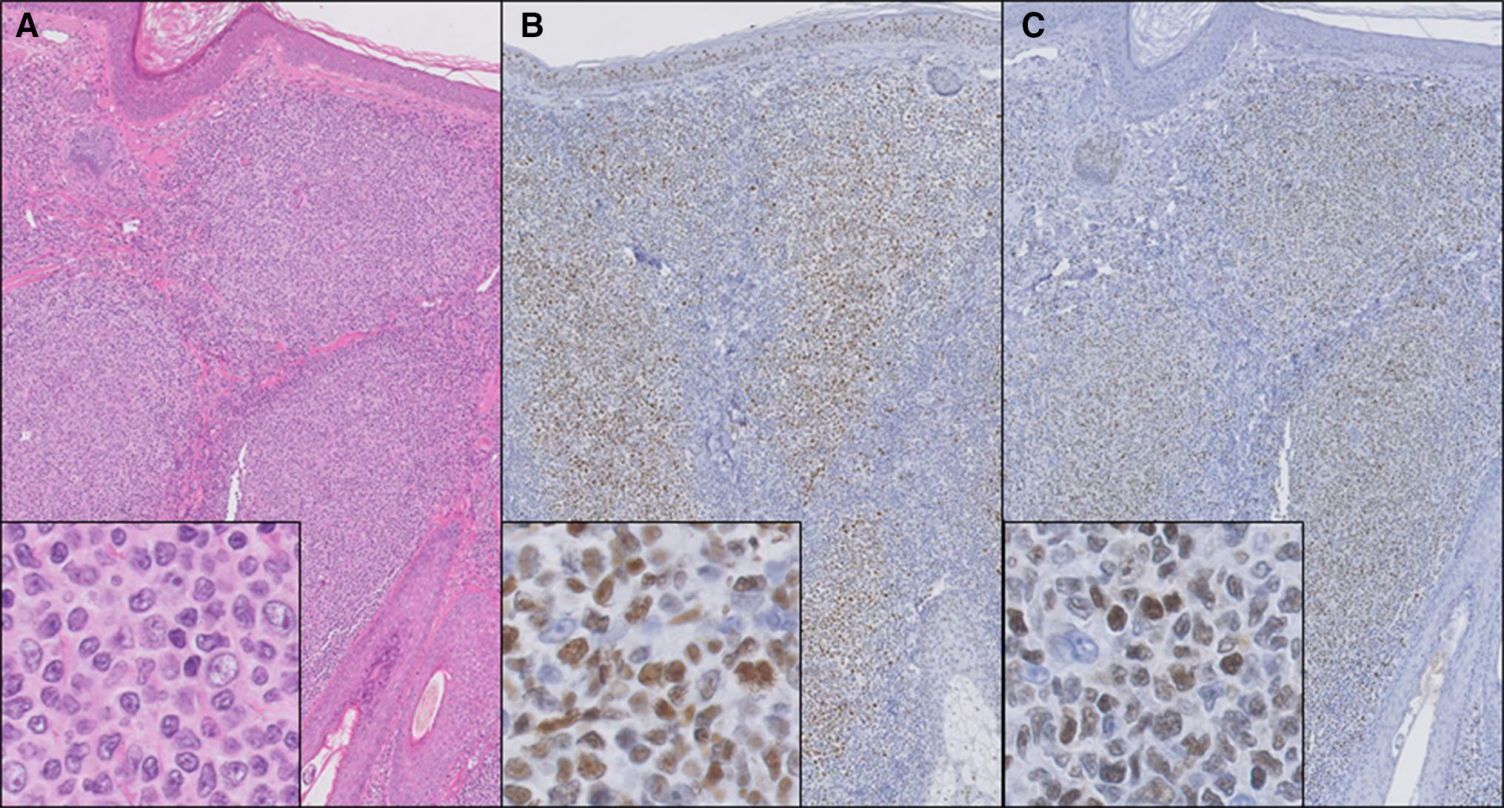
Histopathology of a patient with primary cutaneous follicle center lymphoma with a follicular growth pattern. Hematoxylin–eosin staining (**a**) shows nodular dermal infiltrates of large centrocytes (*insets*), which stain positively for BCL6 (**b**) and TOX (**c**). (**a**–**c** ×100, and *insets* **a**–**c** ×400)

TOX was expressed by the neoplastic follicle center cells in 16/17 patients (94 %) with PCFCL and in 7/7 patients (100 %) with skin lesions of systemic FL (Fig. 2). In these cases, TOX and BCL6 also showed a very similar staining pattern. Remarkably, in one case of PCFCL with a diffuse growth pattern, the neoplastic follicle center cells were positive for BCL6 but completely negative for TOX, while TOX was expressed by scattered small lymphocytes, serving as a useful internal control.

### TOX expression in primary cutaneous marginal zone lymphoma

In none of the five patients with PCMZL, TOX was expressed by the marginal zone B-cells or the plasma cells. In three patients, small reactive follicles were present that showed TOX expression in a pattern as observed in reactive follicles of lymph nodes and tonsils. In addition, TOX was expressed by small scattered lymphocytes.

### TOX expression in primary cutaneous diffuse large B-cell lymphoma, leg type and secondary cutaneous involvement of systemic diffuse large B-cell lymphoma

**Fig. 3:**
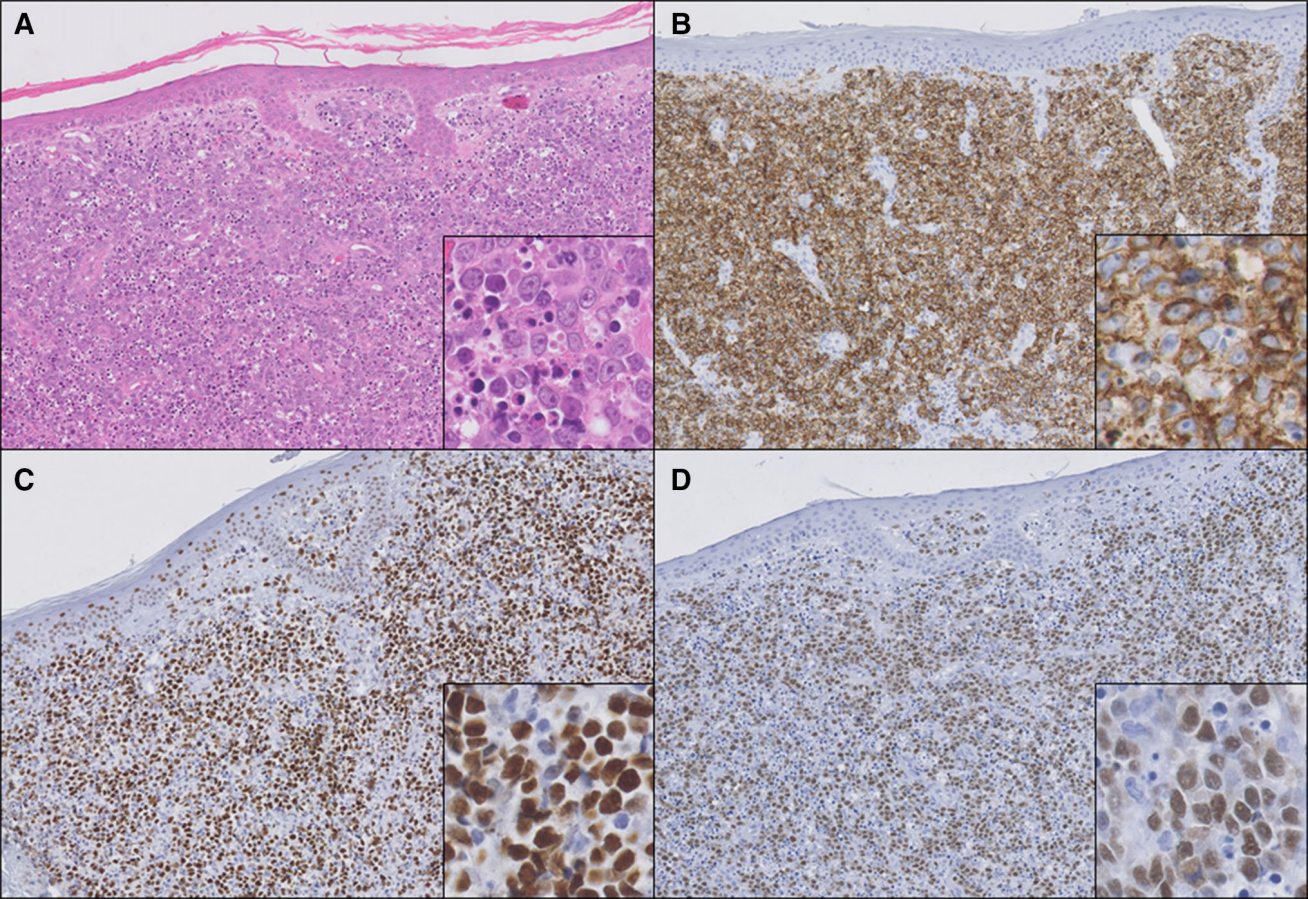
Histopathology of a patient with primary cutaneous diffuse large B-cell lymphoma, leg type. Hematoxylin–eosin staining (**a**) shows diffuse infiltration of the dermis by sheets of CD20^+^ (**b**) centroblasts and immunoblasts with many mitotic and apoptotic figures (*inset* in **a**). These blastic B-cells stain positive for BCL6 (**c**) and TOX (**d**) (**a**–**d** ×100, and *insets* **a**–**d** ×400)

Thirteen patients with PCDLBCL,LT and two patients with secondary cutaneous involvement of systemic DLBCL were evaluated. In general, approximately two-third of the PCDLBCL,LT showed BCL6 expression in more than 50 % of the neoplastic B-cells [12]. In our current series, 10/13 patients (79 %) with PCDLBCL,LT were positive for BCL6. Four of these cases (40 %) expressed TOX in more than 50 % of the neoplastic B-cells (Fig. 3). Intensity was strong in 3/4 cases (75 %) and dim in 1/4 cases (25 %). In the other six patients with a BCL6^+^ PCDLBCL,LT and in the three patients with a BCL6^−^ PCDLBCL,LT, TOX was expressed by a minor proportion of the neoplastic B-cells or was completely negative. The two patients with secondary cutaneous involvement of systemic DLBCL, both BCL6^+^, showed either strong (*n* = 1) or weak (*n* = 1) TOX expression in more than 75 % of the neoplastic B-cells. In the group of patients with PCDLBCL,LT, there was no relationship between expression of TOX and the clinical course of the patients (data not shown).

## Discussion

This study demonstrates that TOX is not only expressed by neoplastic T-cells in CTCL, but also by follicle center cells in all reactive follicles in lymph nodes, tonsils, CLH, and PCMZL, and by neoplastic follicle center cells in 16/17 PCFCL, and 7/7 secondary cutaneous FL. In all these cases, the staining pattern of TOX was very similar to that of the follicle center cell marker BCL6. In addition, TOX was expressed by 4/10 BCL6^+^ PCDLBCL,LT and 2/2 BCL6^+^ secondary cutaneous DLBCL, but not or only weakly in a minority of the neoplastic B-cells in 3/3 BCL6^−^ PCDLBCL,LT.

Currently, the literature on TOX in association with B-cells is scarce. In developing B-cells, no TOX expression was present, and mice deficient in TOX appeared to have normally developed B-cells in the bone marrow and spleen [1, 2]. In addition to the loss of CD4^+^ T-cells, these mice lacked normal development of lymph nodes and Peyer’s patches. However, this was attributed to dysfunction of LTi cells and not to dysfunction of B-cells [2]. Interestingly, one study suggested that TOX is involved in germinal center B-cell development and/or function, referring to a study distinguishing subtypes of DLBCL by genetic profiling, but further details were not provided [3, 9].

In CBCL, TOX expression has only been described to be negative in PCDLBCL,LT, but the number of cases and data on BCL6 expression were not provided [8]. In B-cell lymphomas other than CBCL, TOX deletions, but not expression, were detected in 3/45 patients (7 %) with primary central nervous system lymphomas, and in 2/126 patients (2 %) with acute lymphoblastic leukemia, of which one had a B-precursor phenotype [5, 6, 10].

In conclusion, TOX is expressed not only by both reactive and neoplastic T-cells, but also by both reactive and neoplastic follicle center cells and a proportion BCL6^+^ PCDLBCL,LT and secondary cutaneous BCL6^+^ DLBCL. The functional significance of TOX expression in reactive and neoplastic B-cells remains to be elucidated.
